# Land or sea? Foraging area choice during breeding by an omnivorous gull

**DOI:** 10.1186/s40462-016-0078-5

**Published:** 2016-05-15

**Authors:** Natalie Isaksson, Thomas J. Evans, Judy Shamoun-Baranes, Susanne Åkesson

**Affiliations:** Centre for Animal Movement Research, Department of Biology, Ecology Building, Lund University, Lund, SE-223 62 Sweden; Computational Geo-Ecology, University of Amsterdam, Postbus 94248, Amsterdam, 1090 GE The Netherlands

**Keywords:** Behavioral plasticity, Foraging ecology, Generalist foraging, GPS tracking, Ground-truthing, Habitat use, Individual repeatability, *Larus fuscus*, Lesser black-backed gull

## Abstract

**Background:**

Generalist predators may vary their diet and use of habitat according to both internal state (*e.g.* breeding stage) and external (*e.g.* weather) factors. Lesser black-backed gulls *Larus fuscus* (Linnaeus 1758) are dietary generalists, foraging in both terrestrial and marine habitats during breeding. We investigate what affects the gulls’ propensity to forage at sea or on land. We assess the importance of terrestrial foraging to gulls in the Baltic Sea (sub. sp. *L. f. fuscus*), looking especially at their use of agricultural fields.

**Results:**

Through the GPS tracking of 19 individuals across 3 years we tracked 1038 foraging trips and found that 21.2 % of foraging trips were predominantly terrestrial, 9.0 % were a mix of terrestrial and marine, and 68.5 % were exclusively marine. Terrestrial trips were (1) more frequent when departing around sunrise, whereas marine trips occurred throughout the day. Additionally, trips with mostly land-based foraging decreased as the breeding season progressed, suggesting dietary switching coincident with the onset of chick provisioning. (2) During cloudy and cold conditions terrestrial foraging trips were more likely. (3) We found no differences between sexes in their land-based foraging strategy. (4) Gull individuals showed great variation in foraging strategy. Using observations of agricultural fields, carried out for one year, we found that (5) gulls preferentially foraged on fields with short vegetation, and there was a positive association with occurrence of waders and other species of gulls. (6) The availability and use of these preferred fields decreased through the breeding period.

**Conclusions:**

This study found high prevalence of terrestrial foraging during early breeding as well as support for dietary switching early in the breeding season. The overall tendency for marine or terrestrial foraging was consistent within individuals, with gull identity accounting for much of the variation observed in foraging trips. Our results suggest that anthropogenic terrestrial food sources may play a role in the low breeding success of these gulls through either variation in quantity and/or quality. Finally, our study demonstrates the potential of combining data from GPS-tracking of individual animals with the ‘ground-truthing’ of habitat visited to elucidate the otherwise nebulous behavior of a generalist predator.

**Electronic supplementary material:**

The online version of this article (doi:10.1186/s40462-016-0078-5) contains supplementary material, which is available to authorized users.

## Background

Generalist predators may vary their diet and use of habitat according to both internal state (*e.g.* breeding stage) and external (*e.g.* weather) factors [1, 2]. The breeding season poses particular challenges to individuals that need to balance their own energetic requirements with that of their offspring [3]. Studies that employ long-term GPS tracking of individuals [4] complemented by detailed data on foraging site conditions [5] have the potential to elucidate factors contributing to breeding success and survival of long-lived species.

**Fig. 1:**
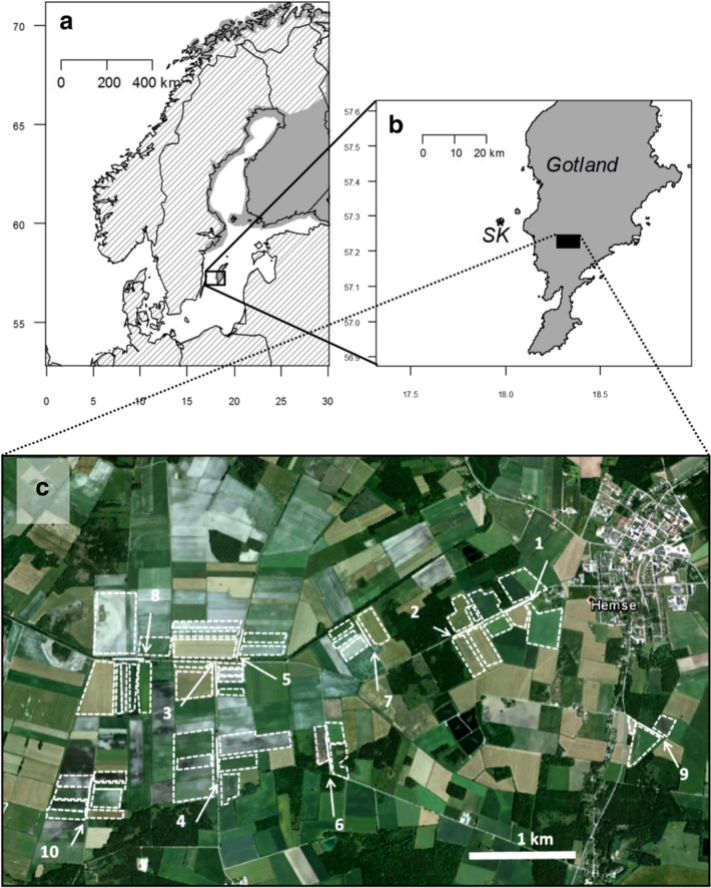
Study area map. Maps showing; (**a**) breeding range of the nominate lesser black-backed gull sub-species (*Larus f. fuscus*), grey uniform fill, after [71]. **b** Expansion showing colony location (SK*), and location of study fields (*black filled rectangle*). **c** Location of field transects (*white lines*) with arrows marking start position (precise location in supplementary table 2) and the bordering fields included in the study indicated (outlined by *dashed white lines*) (Map data: Google, Lantmäteriet/Metria)

*Larus* gulls are an example of generalist predators with omnivorous diet which typically make use of both marine and terrestrial habitats [6, 7]. As generalists, gulls may be expected to use a variety of different food sources throughout the breeding season in order to maximize fitness, depending on physiological requirements, food availability and predictability, energetic costs, and inter- or intra-specific competition. The lesser black-backed gull *Larus fuscus* (Linnaeus 1758) of which the nominate sub-species (*L. fuscus fuscus*) was studied here, uses mostly marine food sources during breeding [8, 9], which they are thought to rely more on than other subspecies (*L. f. graeslii*, *L. f. intermedius*) [10]. At the Karlsö islands (Fig. 1), the largest breeding site of regionally endangered *L. f. fuscus* in the Baltic Sea [11, 12], foraging on agricultural fields is expected to be common during breeding as terrestrial prey items were observed during an earlier study at the colony [13]. Gulls in general are increasingly using anthropogenic food sources including fisheries discards, refuse dumps, and agricultural land [14, 15]. Stable isotope analysis of feathers from gulls in one area taken over several decades suggested increasing reliance on terrestrial food sources likely due to declining fish abundance [16]. In the Baltic Sea cod stocks have plummeted while sprat and herring stocks have increased leading to an ecological regime shift [17]. Though sprat and herring numbers are high, their quality is reduced as a result of density-dependent food competition [18], with potential consequences for breeding seabirds [19]. Agricultural fields are a relatively new food source in the area and could be exploited in the face of declining quantity or quality of previous (more marine) food sources. However, it is not currently well understood how such novel anthropogenic food sources influence foraging patterns and how they may affect population viability in the long term.

During breeding, gulls are central-place foragers, sensitive to the availability of prey in both space and time and modulate their foraging behavior accordingly [6, 20, 21]. Therefore, we can expect lesser black-backed gulls to vary their use of land and sea foraging habitats across the course of a day and between years, depending on the availability of prey. Previous studies on lesser black-backed gulls suggest dietary switching, from terrestrial to marine food sources occurs in conjunction with changing breeding stage, from incubation to chick-rearing [22–24]. During incubation, breeding adults may be selecting for food that is more predictable in time and space as opposed to of high quality [22]. Later in the season, chicks may require high-energy and easily digestible food items, which are more likely to be provided by marine fish than terrestrial invertebrates [15, 23–25]. We may therefore expect gulls at our colony to display similar dietary switching behavior during the course of the breeding season.

Records of foraging on agricultural land by gulls show that earthworms (Lumbricidae) and insects (Insecta) are frequent prey items [26, 27]. These prey are more available under rainy conditions [28], increasing the likelihood of gulls using agricultural fields during cloudy and wet weather. As gulls forage by sight and walk with an upright posture; agricultural fields with short sparse vegetation and fields with crops having these characteristics are likely to be targeted when foraging on land [29]. The availability of these types of fields will both determine field selection by terrestrially foraging gulls, and as field conditions change through the breeding season, the propensity of gulls to choose to forage on land or at sea.

Finally, recent studies suggest individual and sex differences in foraging behavior [7], with these differences potentially arising from sex specific nutritional requirements such as for females during egg-production [30] or from risk partitioning [31]. It can therefore be hypothesized that female lesser black-backed gulls would forage more on land than males and that individuals may show a propensity for one or the other foraging strategy.

In our study we used a combination of GPS tracking during three breeding seasons (2011–2013), and observations on agricultural land during one breeding season (2013) to characterize lesser black-backed gull foraging behavior. We investigated how gulls chose between terrestrial or marine foraging trips, and what factors affected this decision. As agricultural fields are the only significant terrestrial food source for the gulls in this area with limited refuse dump availability [13], we looked at what kinds of agricultural fields gulls use during terrestrial foraging. Specifically we focused on the following questions: (1) how the gulls vary their propensity for terrestrial or marine foraging trips over time, from time of day, across the breeding season, and among years; (2) how weather affects their foraging decisions, (3) whether sexes differ in their foraging behavior, (4) whether individuals are consistent in foraging strategy, (5) what the characteristics are of the agricultural fields that they forage on including any potential association with other foraging bird species, (6) and how the availability of these suitable agricultural fields changes through the breeding period.

## Methods

The study was carried out at Stora Karlsö, Gotland, Sweden (57°17’ N, 17°58’ E), a small island (2.5 km^2^) in the western Baltic Sea, located 7 km to the west of Gotland (Fig. 1), a large island (>3,000 km^2^) with mixed land-use, including forestry and arable agriculture. Stora Karlsö has major seabird breeding populations of common murre *Uria aalge*, razorbill *Alca torda*, great cormorant *Phalacrocorax carbo*, herring gull *Larus argentatus*, arctic tern *Sterna paradisaea*, and lesser black-backed gull (ca. 500 pairs), with smaller populations of other species [32]. Locally, numbers of the eastern breeding nominate subspecies, *L. f. fuscus*, have been declining [11]. On a national level the species has an IUCN regional Red List categorization of endangered [12].

### GPS tracking

During incubation in late May and early June of 2011–2013 we deployed 18 g solar-powered GPS devices with remote-download capability (UvA-BiTS, University of Amsterdam, The Netherlands) [33] on 21 breeding lesser black-backed gulls (see Additional file 1). Devices were attached using harnesses constructed of tubular Teflon™ ribbon (Bally Ribbon Mills 8476-.25") with a 1 mm braided nylon wader shelf string (British Trust for Ornithology) inserted, either with a wing- (2011) or full body- harness (2012, 2013) (see [34] for description of harness types). Attachment method changed between years in order to improve re-capture rates, however we recommend the wing harness following [34]. The combined weight of the GPS device plus harness was ca. 21 g, corresponding to a mean of 2.9 % (range 2.3 – 3.6 %) of body mass at time of deployment. GPS tracking was continuous with location intervals typically of 300 or 600 s, though with some gaps following prolonged overcast conditions. Data were downloaded and programs uploaded to the GPS devices when the gulls were present at the breeding colony, where a network of 2–4 antennas provided good coverage of the breeding area. Birds were sexed morphologically by head plus bill measurements according to [35], with a colony-specific discriminant threshold of 113.5 mm (< female, > males).

Foraging trips during the breeding season (May-July) were analyzed, focusing on colony-wide breeding stages of 20 days each: incubation (May 20-June 9), early chick-rearing (June 10–30) and late chick-rearing (July 1–21). Pre-laying stage (April 30- May 19) data from 2012–13 were used in further analyses of most competitive models, in order to minimize the effect of lack of data from 2011. GPS point data were segmented by setting a distance threshold (500 m) around each gull’s nest, defining the start (>500 m) and end of trips (<500 m). Trips were filtered to include only normal breeding period foraging trips (‘long trips’ in [7]) with adequate GPS tracking data for analysis (see Additional file 2).

To analyze the tendency of gulls to use land or sea habitat during the GPS tracked foraging trips, we classified the proportion of foraging time spent on land (for full details see Additional file 2). To do this, within foraging trips, likely foraging GPS locations were extracted for each trip using two speed thresholds and a minimum distance of 3 km from the colony. Then these foraging locations were classified as either on land or at sea. Finally for our response variable for statistical analyses, for each trip we calculated the proportion of foraging locations on land rather than sea, a value between 0 (all foraging locations at sea) and 1 (all foraging locations on land). The foraging trip data were further annotated with weather information, departure time relative to sunrise time, breeding stage (see above), year (2011, 2012, 2013), and with the proportion of terrestrial and marine foraging (see below for more details). Weather data were extracted and summarized for the colony location (Stora Karlsö) for the 24 h period up to the departure time of each foraging trip (see Additional file 3). These were extracted from ‘NCEP-DOE Reanalysis 2’ [36] using R [37] package *RNCEP* [38]. The variables extracted were: precipitation rate, air temperature at 2 m, east and north wind vectors at 10 m, total cloud cover (*RNCEP* variable names, respectively: *prate.sfc, air.2 m, uwnd.10 m*, *vwnd.10 m* and *tcdc.eatm*), which were linearly interpolated to the colony location and time points: (relative to departure time *t*_*dep*_) *t*_*dep*_, *t*_*dep*_*-6 h*, *t*_*dep*_*-12 h*, *t*_*dep*_*-18 h.* Mean values were then calculated from the 4 time points for all variables except precipitation rate, where the sum was taken (representing cumulative rainfall during 24 h preceding trip departure time). We analyzed wind speed in both N-S and E-W components, with E-W corresponding approximately to head/tail winds when travelling to and from Gotland, and N-S to side-winds if travelling to/from Gotland. We calculated a variable ‘sunrise proximity’, to indicate how close to sunrise a trip departure occurred. Sunrise times for the day of each foraging trip were extracted with function sunriset in R package *maptools* [39] this was then transformed by taking the cosine of time-since-sunrise divided by 12 and multiplied by π, i.e. cos(π*time-since-sunrise/12) following a similar calculation by [40]. This provided a value between 1 and −1, with values at: sunrise, 1; 6 h from sunrise, 0; and 12 h, −1. The proportion of foraging time spent on land (see Additional file 2 for details) was used as the response variable (see Statistical Analysis below). For illustration this was put into three classes: sea (<5 % foraging time on land), mixed (5–95 % foraging time on land), and land (>95 % foraging time on land).

### Agricultural fields

A ca 28 km^2^ agricultural area in south-west Gotland with representative fields of the region was selected as a field site (Fig. 1 b). Site selection was based on the activity of GPS-tagged lesser black-backed gulls in the period just prior to the study period (late May 2013). Within this area we plotted ten transects along roadways, each of 300 m in length (Fig. 1 c). Transects were selected to include at least one field visited by a GPS tracked gull, plus at least one control field where gulls had not been recorded. Fields bordering each transect were surveyed giving a total of 50 fields (see Additional file 4). Of these, seven fields were visited by two GPS-tagged individuals (individuals 8114314 and 8111250) during the study period. Fields were surveyed during June and early July 2013, representing late incubation or early chick-rearing through to middle-late chick-rearing in our colony of lesser black-backed gulls (pers. obs.). Fields were visited during three 6-day periods: 13–18 June, 24–29 June, and 3–8 July. During each period we observed in the morning (04:00–10:00 h, days 1–3) and evening (16:00–22:00 h, days 4–6). Over a twenty minute period at each field we counted the maximum numbers of the following species present: black-headed gull, common gull *L. canus* (Linnaeus 1758), herring gull, lesser black-backed gull, lapwing *Vanellus vanellus* (Linnaeus 1758), and oystercatcher *Haematopus ostralegus* (Linnaeus 1758). As we were interested in general characteristics of fields typically used and those seemingly avoided by gulls, we did not make a distinction between age classes or behaviors in our observations but noted presence or absence. In our observations birds regularly fed with bouts of foraging (e.g. active searching for prey where they were walking with head down) interspersed with resting. Thus it was likely that presence on a field was a strong indication that the bird was present for foraging, even if active foraging was not observed within the twenty minute observation period. Earthworm availability on the fields was also assessed. We walked along a 4x25 m gate transect at a speed of one footstep per second and counted how many earthworms were seen within a 1 m perimeter, similar to the method described in [41].

To assess the vegetation characteristics of the fields and how these changed through the season we surveyed each field during each of the three observation periods, visiting during non-observation hours (i.e. 10:00–16:00). To account for variation within fields we placed five quadrats (1x1 m) each 3 m from the field border ensuring that at least three of the four or more sides of fields were covered. For further analysis we took the mean for each field and period of vegetation height and percentage vegetation cover (consequently ranked into 4 classes: 0-25 %, 25-50 %, 50-75 %, and 75-100 %). During field visits the crop plants grown in fields were identified. We pooled crops into 5 crop types: cereal (barley, oats, wheat), grass (grasses), roots (carrots, potato), ryegrass, and other (rapeseed, snow pea).

### Statistical analyses

Two sets of statistical analyses were made to look at the foraging activity by the lesser black-backed gulls, first on their use of terrestrial over marine foraging within GPS tracked foraging trips and secondly to look at what fields were used by gulls and when. For the GPS tracked foraging trip analysis the proportion of terrestrial foraging during each trip was the response variable. For the field observation analysis, the presence/absence of lesser black-backed gulls was the binomial response variable. This binomial variable was chosen as very few lesser black-backed gulls were observed during the field observations thus these observations were reduced to presence or absence. In order to model the probability of marine or terrestrial foraging during trips we constructed a priori logistic mixed effects models with individual gull identity included as the random effect. Field observations included field ID nested within transect ID as a random effect. Generalized linear mixed models (GLMMs) were built with the package *lme4* [42] in R, using the *glmer()* call and specifying family as binomial and link as logit. Final models were selected based on lowest corrected Akaike Information Criterion (AICc) values [43]. The proportion of total variance explained by fixed effects in models was assessed using marginal R^2^ values, and that explained by the combined fixed and random effects by conditional R^2^ values [44] extracted with the *R* package *MuMIn* [45]. The most competitive models for GPS tracked foraging trips were re-run with the dataset including data from the pre-laying period in 2012–2013. In the final selected models we tested for variable significance by dropping single terms then comparing these to the full model via likelihood ratio tests. The final model was standardized [46] using the R package *arm* [47] and assessed for collinearity, with a kappa value of less than 10 considered acceptable [48].

We quantified how consistent the GPS tracked gulls were in their tendency to use terrestrial or marine habitat during foraging trips by calculating repeatability (*r*), as the proportion of the total variance in the response variable (i.e. proportion of foraging locations with each trip spent on land rather than at sea) accounted for by differences between individuals rather than within individuals. The result was a value between 0 (all variation is within rather than between individuals) and 1 (all variation is explained by differences between individuals) [49]. We obtained *r* for the standardized best fit model (thus repeatability once main effects are accounted for) and calculated a p value by randomization and the 95 % confidence intervals from a parametric bootstrap, both with 1000 iterations (following method of [49] as outlined in [50]).

## Results

### GPS tracking

**Fig. 2:**
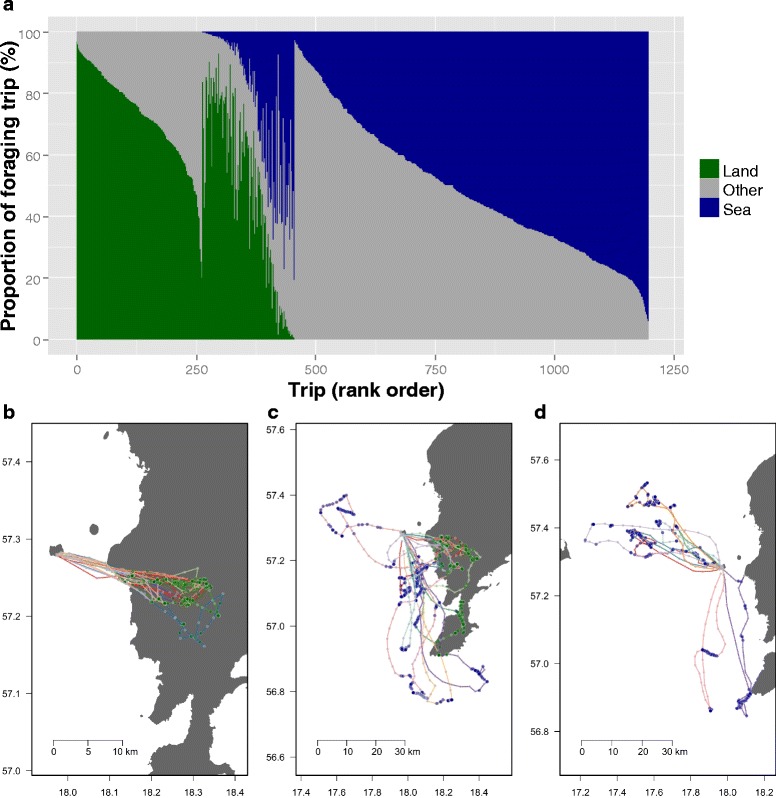
GPS tracked foraging trips classified according to time spent foraging on land and sea. **a** Time spent foraging on land (*green*) or sea (*blue*) or ‘other’ (*gray*, likely non-foraging activity). Trips are ranked in reverse order from most terrestrial to least terrestrial, with the ranking according to proportion of foraging time on land, then the proportion of the whole trip on land, then by the proportion of the whole trip at sea. Notice ‘mixed’ class trips (ca. ranks 250 – 450) with both terrestrial and marine foraging, though the majority trips include foraging exclusively on land (ca. ranks 0 – 250) or sea (ca. ranks 450–1200). 8 example trips (GPS tracks) are shown for those with >95 % foraging time on land (**b**), 5–95 % foraging time on land (**c**), and <5 % foraging time on land (**d**). **b**-**d** points show interpolated GPS locations (see methods), with locations colored to indicate non-foraging points (*gray*), sea foraging (*blue*), and land foraging (*green*); individual foraging trip trajectories are indicated by unique line colors, and land is shown (*dark gray*)

We obtained GPS tracking data for 21 lesser black-backed gulls during the period 2011–2013 (see Additional file 1). However, for one individual the device failed, and the other abandoned breeding shortly after capture, thus both individuals were excluded from further analyses. This resulted in a dataset of 1038 foraging trips (examples in Fig. 2 and overall distribution given in Additional file 5) from 19 individuals (see Additional file 3) for the focal period (20th May – 21st July). Of these foraging trips 220 (21.2 %) consisted entirely of terrestrial foraging, 93 (9.0 %) were a mix of terrestrial and marine foraging, and 711 (68.5 %) were exclusively marine foraging (14 trips were unclassified, see Additional file 2). Foraging trips had a median duration of 4.8 h (IQR 2.6-8.6) with median maximum distance of 22.3 km (IQR 15.3-37.5) from the colony, though with considerable variation (see also, Additional file 2: Figure S1 and Figure S2 respectively, and Additional file 5). These parameters also varied between trip types, with median durations of 6.2 h (IQR 4.1-9.8), 3.9 h (IQR 2.2-6.8), and 11.3 h (IQR 8.1-15.0) for land, sea, and mixed trips respectively. Median maximum distances were 20.8 km (IQR 17.4-22.7), 23.2 km (IQR 13.2-41.1), and 39.3 km (IQR 22.6-49.3) for land, sea, and mixed trips respectively.

**Fig. 3:**
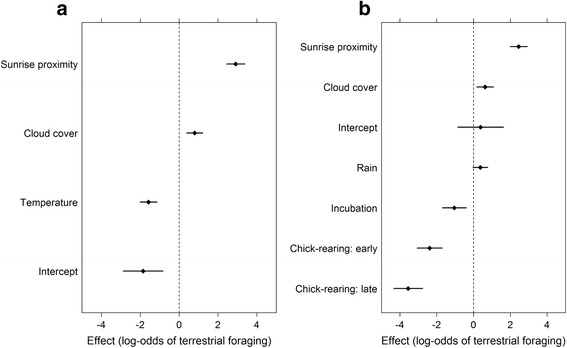
Factors affecting the probability of GPS tracked lesser black-backed gulls to forage on land or at sea for two statistical models: of all years (2011–2013) for incubation through to late chick-rearing (**a**), and for 2012–2013 with the addition of the pre-laying period **b**. Main effects terms for models showing the factors affecting the probability of GPS tracked lesser black-backed gulls performing terrestrial rather than marine foraging trips. Coefficients and 95 % confidence intervals (Wald estimates) are shown for the standardized models allowing direct comparison of effects between variables. Coefficients are compared to the model reference level: incubation stage 2011 (**a**), pre-laying stage 2012 (**b**)

**Fig. 4:**
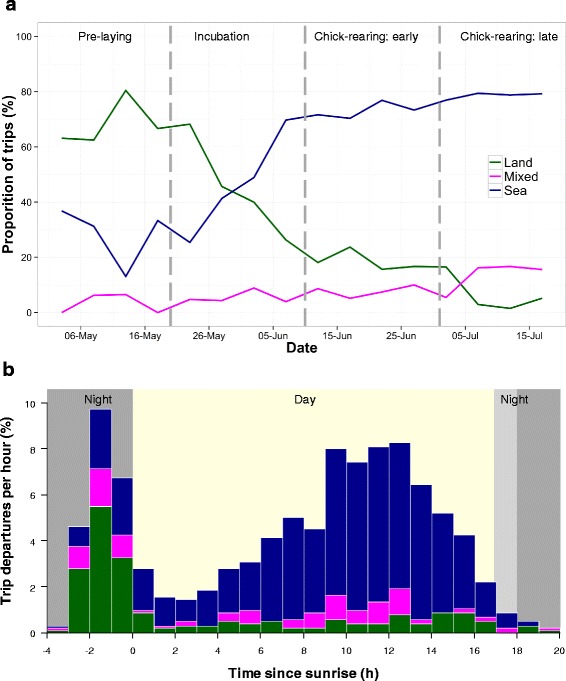
The proportion of foraging trips by GPS tracked gulls classified as land, sea, or mixed for the breeding period (**a**) and per hour based on time of departure (**b**). **a** For each 5 day period from all 3 years pooled (also see Additional file 8 where years are separated). The breeding stages are indicated (*vertical broken grey lines*). Note that the pre-laying period was calculated from 2012 & 2013 only as data for 2011 were unavailable. **b** Proportion of all foraging trip departures occurring each hour relative to time of sunrise performed by the gulls with data pooled for all breeding stages and years. Trip types are indicated (following same colors as panel a). Period of day is indicated, with night (*dark grey*), and day (*light yellow*); as the length of night was not constant throughout the study the maximum- (*light grey*) and minimum- (*dark grey*) night duration are indicated

The propensity for lesser black-backed gulls to use terrestrial rather than marine habitat during foraging trips was most affected by trip departure time relative to sunrise time, cloud cover and temperature (d.f. = 3, *χ*2 = 289.8, model 18 in Table 1, Fig. 3a). Terrestrial foraging predominated on foraging trips departing soon before or after sunrise (Fig. 4 b, *χ*2 = 213.9, *p* < 0.001). Weather also affected the propensity for terrestrial foraging, with greater cloud cover (*χ*2 = 15.2, *p* <0.001) and lower temperatures (*χ*2 = 56.4, *p* < 0.01) increasing the likelihood. There was no difference between male and female gulls in their tendency to perform terrestrial foraging trips, with models containing sex having higher AICc (e.g. models 8–10 in Table 1, Additional file 6). However, we did find that including individual differences greatly improved the model fit, with individual included as a random effect leading to a high conditional R^2^ (0.698), much higher than the marginal (main effects) R^2^ (0.280). This was further corroborated by analysis of individual repeatability, which was significant and very high (*r* = 0.814, *p* < 0.001, 95 % CI = 0.612 - 0.891).

**Table 1:** Summary statistics for GLMMs to look at temporal and weather influences on the probability of lesser black-backed gulls (*n* = 19) to forage on terrestrial- over marine- habitat during foraging trips (*n* = 1024). All models include individual gull ID as a random effect. Akaike information criterion adjusted for small sample sizes (AICc), change in AICc relative to the best-fit model (18) and marginal R^2^ (R^2^m) and conditional R^2^ (R^2^c) values are presented

	Model	d.f.	AICc	∆AICc	R^2^m	R^2^c
1	intercept	2	974.6	283.8	-	0.472
2	cloud + temp	4	902.7	211.9	0.086	0.503
3	cloud + temp + ppt	5	899.1	208.2	0.093	0.509
4	stage + cloud + temp + ppt + temp*stage	9	893.6	202.7	0.119	0.506
5	stage + cloud + temp + ppt	7	891.0	200.2	0.118	0.509
6	sunrise_prox	3	753.2	62.35	0.195	0.670
7	stage + sunrise_prox	5	713.9	23.07	0.265	0.672
8	stage + cloud + temp + ppt + sunrise_prox + year + windNS + windEW + sex + temp*stage + windNS*windEW	16	704.1	13.27	0.297	0.692
9	stage + cloud + temp + ppt + sunrise_prox + year + windNS + windEW + sex + temp*stage	15	702.1	11.20	0.291	0.702
10	stage + cloud + temp + ppt + sunrise_prox + year + windNS + windEW + sex	13	699.5	8.593	0.285	0.704
11	stage + cloud + temp + ppt + sunrise_prox + year + windNS + windEW	12	697.4	6.566	0.289	0.703
12	stage + cloud + temp + ppt + sunrise_prox + year + temp*stage	12	697.1	6.226	0.284	0.697
13	stage + cloud + temp + ppt + sunrise_prox + year	10	694.5	3.655	0.284	0.702
14	stage + cloud + temp + ppt + sunrise_prox + temp*stage	10	694.1	3.233	0.298	0.693
15	stage + cloud + temp + ppt + sunrise_prox	8	692.1	1.236	0.292	0.697
16	cloud + temp + ppt + sunrise_prox	6	691.4	0.573	0.282	0.699
17	stage + cloud + temp + sunrise_prox	7	691.4	0.554	0.290	0.695
**18**	**cloud + temp + sunrise_prox**	**5**	**690.9**	**0.000**	**0.280**	**0.698**

cloud = cloud cover(%); ppt = precipitation, stage = incubation, early chick-rearing, late chick-rearing; sex = individual sex (female, male); temp = air temperature; sunrise_prox = proximity to sunrise; year = year (2011, 2012, 2013); windNS = wind vector in North–south direction; windEW = wind vector in East–west direction

Selected model (18) is indicated in bold

The most competitive models (15–18 in Table 1), with ∆AICc < 2, were re-run with the dataset from 2012–2013 that also included data on the pre-laying period (April 30-May 20). The final model (model 6 in Table 2, κ = 5.79) was selected based both on AICc value and a threshold of κ < 10, with the addition of temperature (model 7 in Table 2) slightly reducing AICc but leading to an unacceptable increase in model collinearity (κ = 14.4). Here, the propensity for lesser black-backed gulls to use terrestrial rather than marine habitat during foraging trips was most affected by trip departure time relative to sunrise time, breeding stage, cloud cover and precipitation (d.f. = 6, *χ*2 = 282.1, model 6 in Table 2, Fig. 3b). Terrestrial foraging predominated on foraging trips departing soon before or after sunrise (*χ*2 = 138.7, *p* < 0.001) and under cloudier (*χ*2 = 7.601, *p* < 0.01) conditions. Rainier (*χ*2 = 3.355, NS, *p* = 0.067) conditions may also favor terrestrial foraging, with inclusion of this term marginally lowering AICc (Table 2), but the term itself was not significant at *p* < 0.05. Breeding stage was an important driver of foraging strategy, with fewer terrestrial trips occurring as the season progressed (*χ*2 = 105.7, *p* < 0.001).

**Table 2:** Summary statistics for GLMMs to look at temporal and weather influences on the probability of lesser black-backed gulls (*n* = 14) to forage on terrestrial- over marine- habitat during foraging trips (*n* = 959), including the pre-laying period (April 30 - May 19) in 2012 and 2013. All models include individual gull ID as a random effect. Akaike information criterion adjusted for small sample sizes (AICc), change in AICc relative to the best-fit model (7) and marginal R^2^ (R^2^m) and conditional R^2^ (R^2^c) values are presented

	Model	d.f.	AICc	∆AICc	R^2^m	R^2^c
1	Intercept	2	939.0	270.5	-	0.491
2	cloud + ppt + sunrise_prox	5	768.6	100.0	0.162	0.664
3	cloud + temp + ppt + sunrise_prox	6	677.3	8.745	0.311	0.692
4	cloud + temp + sunrise_prox	5	677.0	8.411	0.308	0.691
5	stage + cloud + temp + sunrise_prox	8	669.5	0.959	0.330	0.698
**6**	**stage + cloud + ppt + sunrise_prox**	**8**	**669.0**	**0.459**	**0.328**	**0.698**
7	stage + cloud + temp + ppt + sunrise_prox	9	668.6	0.000	0.336	0.699

Variable names as in Table 1

Selected model (6) is indicated in bold

### Agricultural fields

**Fig. 5:**
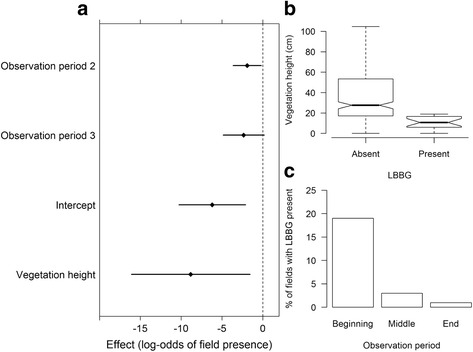
Main effects of gulls’ presence on agricultural fields 2013. **a** Main effects terms for model 9 (Table 2), showing the factors affecting the probability of lesser black-backed gulls being present on agricultural fields during the study period in 2013. Coefficients and 95 % confidence intervals (Wald estimates) are shown for the standardized model allowing direct comparison of effects between variables. These are compared to study period 1 during 2013. **b** Boxplot showing the negative relationship between vegetation height and lesser black-backed gull presence on agricultural fields during the observation period in 2013. Boxplot shows medians (*horizontal line*), approximate 95 % confidence intervals of medians (*notches*), 25th and 75th percentiles (*boxes*), the most extreme data point that is no more than 1.5 times the length of the box away from the box (*whiskers*), and outlying observations beyond these (*circles*). **c** Bar chart showing the decrease in gull presence on agricultural fields during the observation periods (*beginning, middle, end*) in 2013

Data collected on agricultural fields is summarized in Additional file 7. Of the 10 models testing the effect of crop characteristics and time on lesser black-backed gull presence on agricultural fields, the model retaining vegetation height and observation period was the best fit (d.f. = 6, *χ*2 = 40.925, *p* < 0.001, Fig. 5a, model 9 in Table 3). Gulls were more likely to be present on fields with low vegetation height (*χ*2 = 10.81, *p* = 0.001, Fig. 5b) and less likely to visit fields as the study period progressed (*χ*2 = 7.4697, *p* < 0.05, Fig. 5c). The best fit model (model 9, Table 3) included both vegetation height and observation period, which had a lower AICc value than either model including only one of these two variables (models 7 and 8, Table 3). While median vegetation height did increase across observation periods, the variation in vegetation height was high within all periods, thus collinearity in the final model including both variables was not an issue (κ < 10). We tested for associations between lesser black-backed gulls and the number of other birds or earthworms by adding these variables to the best fit model (models 10, 11 and 12 in Table 3). Lesser black-backed gulls were positively associated with both waders (*χ*2 = 4.9, *p* < 0.05) and other gulls (*χ*2 = 25.7, *p* < 0.001), but not with earthworms (*χ*2 = 0.01, *p* > 0.05).

**Table 3:** Summary statistics for GLMMs to look at field characteristics and temporal influences on the probability of lesser black-backed gulls being present on agricultural fields (observations = 300, n fields = 50, n transects = 10). We then tested for associations between lesser black-backed gulls and the number of birds of -other gull species (all gulls excluding lesser black-backed gulls), −waders (lapwings and oystercatchers), and the number of earthworms; by including these terms along with the best fit model (9). All models included field nested within transect as a random effect. Akaike information criterion adjusted for small sample sizes (AICc), change in AICc relative to the best-fit model (9) and marginal R^2^ (R^2^m) and conditional R^2^ (R^2^c) values are presented

	Model	d.f.	AICc	∆AICc	R^2^m	R^2^c
1	day	4	144.1	36.5	0.002	0.55
2	intercept	3	142.3	34.7	-	0.54
3	crop + veg.height + veg.cover	11	121.9	14.4	0.82	0.95
4	crop + veg.height	8	118.1	10.5	0.86	0.95
5	obs.per + day	6	117.9	10.3	0.11	0.92
6	veg.height + veg.cover	7	117.1	9.52	0.83	0.94
7	obs.per	5	116.3	8.73	0.11	0.92
8	veg.height	4	110.9	3.32	0.83	0.94
9	**veg.height + obs.per**	**6**	**107.6**	**0.00**	**0.74**	**0.89**
10	model 9 + earthworm	4	109.7	2.09	0.74	0.89
11	model 9 + waders	4	104.8	−2.80	0.77	0.88
12	model 9 + gulls	4	83.9	−23.6	0.77	0.96

crop = crop type, day = morning/evening, earthworm = number of earthworms, gulls = number of other gulls, obs.per = observation period, veg.cover = vegetation cover, veg.height = vegetation height, waders = number of waders (lapwing and oystercatcher)

Selected model (9) is indicated in bold

## Discussion

By tracking lesser black-backed gulls with GPS over three consecutive years together with observations on agricultural fields we set out to elucidate how these gulls make foraging decisions during the breeding season. We found that: (1) 21.2 % of foraging trips were terrestrial in nature, 9.0 % were a mix of terrestrial and marine, and 68.5 % were exclusively marine; most often gulls departed on terrestrial foraging trips shortly before sunrise, while marine foraging trips occurred throughout the day. A switch in foraging strategy likely occurred during incubation, from terrestrial to marine. (2) Gulls were more likely to forage on land during cloudy and cold conditions. (3) There was no difference between females and males in their land-based foraging behaviour. (4) However, there was evidence for individual differences and consistency in terrestrial foraging. (5) Gulls were attracted to agricultural fields with short vegetation, and there was a positive association with waders and other gull species when on those fields. (6) Lastly, the availability of fields with short vegetation decreased through the season.

During the pre-laying stage (Additional file 8: Figure S4), terrestrial foraging was very high (>50 % of foraging trips). This level of terrestrial foraging during the breeding season contrasts with other populations of lesser black-backed gull in northern Europe, which forage more at sea (e.g. [5, 8, 51]). Precedents for this level of terrestrial foraging can however be found in *L. f. graellsii* (Brehm 1857, the western sub-species) populations from Britain [23, 26]. A recent study employing GPS tracks of lesser black-backed gulls breeding in the North Sea also found increased use of terrestrial foraging sites [52]. However, none of these studies included data from the pre-laying stage. Our long term tracking enables us to explore changes in foraging behavior over all stages of the breeding season, including the pre-laying phase, which is often missed in short term tracking studies, providing new insight into seasonal transitions in foraging strategies. Whether this is a pattern which was missed in previous studies due to lack of higher resolution data or shows a real shift in this species’ behavior as a response to changes in either the terrestrial or marine environments remains to be seen.

The peak departure time for terrestrial foraging trips, the 2–3 h before sunrise and the first hour after sunrise (Fig. 4 b), reflect the expected activity patterns of soil invertebrates [28] and corroborate that the gulls are sensitive to time-dependent prey availability [6, 53]. Terrestrial foraging decreased as the breeding season progressed (Fig. 4 a, Additional file 8), corroborating evidence from other lesser black-backed gull studies which show dietary switching, from terrestrial to marine food sources, in conjunction with the onset of chick rearing [22–24]. Earlier in the season (i.e. May) breeding adults are in the egg-laying and incubation phases and thus may be selecting for food that is more predictable in time and space than marine prey [22, 53]. Later in the season the chicks may need to be fed on high energy content and easily digestible food items, which marine fish are more likely to be than terrestrial invertebrates [15, 23–25]. It has previously been found that intrinsic factors such as breeding stage are the main cause of behavioral changes in foraging [54]. Frequency of mowing and sowing on agricultural fields during the early part of the breeding season, activities with which birds are associated [27, 29], may also play a part. Finally, travel costs may influence the gulls’ foraging location choice [40, 55, 56]. Though the median maximum foraging trip distance for land was shorter than that for sea trips (20.8 km versus 23.2 km), terrestrial foraging trips were of longer duration than those to sea (6.2 h versus 3.9 h). This suggests that while foraging areas on land might be closer to the colony, foraging there takes longer, resulting in a potential trade-off between travel cost and foraging efficiency which contrast between foraging on land or sea. Gulls foraging on land may also spend more time resting, thus contributing to longer durations of terrestrial trips.

Terrestrial foraging trips occurred more frequently when there was greater cloud cover, rainier conditions, and lower air temperatures. Cold and wet conditions have previously been associated with greater soil invertebrate activity [28] as well as with more terrestrial feeding by Hartlaub’s gulls *L. hartlaubii* (Bruch 1853) and common gulls [57, 58]. Unexpectedly, wind did not have a significant effect on foraging area choice, despite wind being a key driver of movement ecology in seabirds [59, 60]. In the study area south-westerly winds predominate, thus gulls may give little weight to wind conditions when making foraging decisions at this colony.

Previous studies on lesser black-backed gulls have found sexual segregation in foraging behavior [7] during incubation and chick rearing, which has also been described in other seabirds such as Northern gannet *Morus bassanus* [61], Audouin’s gull *L. audouinii* [15] and herring gull [62]. We expected that females would forage more frequently on land than males, either due to sex-specific nutrient requirements or risk partitioning [30, 31]; however, we found no such difference (Additional file 6). A recent study on Ring-billed gull *L. delawarensis* (Ord 1815) also did not find sexual segregation in foraging behavior [63]. It could be that during incubation both sexes are foraging more frequently on land because the resources there are more predictable than at sea, thus following a risk-adverse strategy [7, 22, 53].

Individual gulls differed consistently in their propensity to perform terrestrial over marine foraging trips with individual repeatability very high. This is consistent with findings in previous studies on individual specialization in foraging lesser black-backed gulls [7, 20] and has also been documented in herring and Western gulls [64, 65]. Individual specialization in this population might come about through differences in body morphology, experience, and personality. An ultimate explanation may be that variation reduces intra-specific competition for resources and, especially during the breeding season, may increase individual reproductive success through risk partitioning [66]. Our findings potentially suggest that population level generalism in this species may arise through varying levels of individual specialization on different food sources, rather than all individuals being broadly generalists [67].

In addition to the GPS tracking data, our observations on agricultural fields in 2013 revealed that lesser black-backed gulls were present on fields with vegetation shorter than ca. 20 cm (Fig. 5 b). This is consistent with their morphology, as gulls need to be able to walk and see sufficiently in order to forage on fields and this becomes more difficult with higher vegetation [27, 63]. While there was no significant association between earthworms and lesser black-backed gulls it is important to note that the gulls could be foraging more on other invertebrates (e.g., beetles), plant material, or even small mammals, all of which have been found to be important food sources in these gulls [10, 21, 26, 68]. The significant positive relationship between lesser black-backed gulls and other species of gulls and waders suggests that one group (e.g., lapwings, black-headed gulls) could be attracting the others and therefore operating as an indicator [69] or that several gull species favor the same terrestrial foraging conditions [5], explanations which are not mutually exclusive. Of all the temporal variables, only observation period was relevant to lesser black-backed gull presence on agricultural fields (model 7, Table 3), with progressively fewer gulls present later in the season (Fig. 5c). The presence of observation period in the best fit model supports the hypothesis of dietary switching in lesser black-backed gull, especially when the pre-laying period is taken into account (Fig. 4a).

The lesser black-backed gull population on Stora Karlsö has had low breeding success since the mid-2000’s [11, 70], with the reasons behind this unclear. While we may expect generalists to do well, it is possible that due to specializations, parts of the population may suffer under specific changes. A decline in the population of glaucous-winged gulls *L. glaucescens* has for instance been attributed to a switch in diet from marine to terrestrial food sources [15], while conversely yellow-legged gulls *L. michahellis* have benefited from a greater availability of anthropogenic food sources [14]. In light of the importance of terrestrial foraging to this population early in the breeding season, some explanatory factors may be suggested. It may be that anthropogenic activity on agricultural fields in May influences either the quantity or quality of foraging opportunities. Previous studies show that *Larus* gulls are sensitive to spatial and temporal variation in human food resource availability [6, 20, 53]. For gulls, it has been proposed that food foraged on land is of lower quality than that foraged at sea (Nina O’Hanlon., unpublished data; [7]), so while it may be more predictable it is essentially ‘junk food’ that may lower breeding success by decreasing parental body condition. It is noteworthy that terrestrial foraging was highest during the pre-laying period (Fig. 4a), where diet is known to be critical to egg-building in lesser black-backed gulls [30]. The heavy use of terrestrial resources early in the season might also reflect low marine prey quality and/or quantity in the area [17, 18]. Coupled with likely competition with other seabirds at the colony (e.g. common murres and razorbills) that are provisioning chicks with the same prey during this time [19] it may be reasonable to predict that foraging success is too low to provision chicks adequately when the gulls do switch to marine prey. Therefore, terrestrial foraging at this colony may be a symptom of a greater regime shift in the ecosystem, which could help explain low breeding success. Further research and data on individual breeding success would be needed to tease apart these relationships, however.

## Conclusions

We combined the GPS-tracking of individual gulls with ‘ground-truthing’ of habitat visited to elucidate the otherwise nebulous foraging behavior of a generalist predator, the lesser black-backed gull. Our results demonstrate the high prevalence of terrestrial foraging during the early part of the breeding period, with marine foraging most prevalent later in the season. Individual gulls were consistent in their preference for marine or terrestrial foraging suggesting individual specialization. Within a day gulls were more likely to forage on land early in the morning and under cloudy and cold conditions; conditions and a time when prey such as earthworms are most likely to be available on the agricultural fields which the gulls use when on land. Gulls had a preference for fields with low vegetation, which are less available later in the season. That gulls varied their tendency for terrestrial foraging both within days and across the season suggests sophisticated dietary switching driven by some combination of internal state (e.g. breeding stage) and external factors (e.g. weather, time of day, and changes in habitat). This population of gulls has had low breeding success for a number of years; thus our results suggest that this may, in part, be due to variation in quantity and/or quality of anthropogenic terrestrial food sources. Overall the study demonstrates the power of combining individual tracking with ‘ground-truthing’ when studying the foraging behavior of generalist predators.

## Availability of supporting data

The data sets supporting the results of this article are included within the article (and its additional files).

## Additional files

Additional file 1: Table of lesser black-backed gulls GPS deployments (.doc). GPS deployments on lesser black-backed gulls at Stora Karlsö (2011–2013) with number of foraging trips per individual per year included in analysis. (PDF 125 kb) Additional file 2: Detailed analytical methods on the processing of the GPS tracking data of the lesser black-backed gulls (.pdf). Information on which trips were included from the wider GPS tracking dataset and how activity within trips was classified between land and sea foraging is provided. (PDF 304 kb) Additional file 3: Table of analyzed GPS data (.xls). Foraging trips of lesser black-backed gulls, tracked by GPS. The analyzed data are provided as an Excel file, with two sheets, ‘GPS_foraging_trip_data’ giving the analyzed variables, and ‘Metadata’ explaining these variables. (XLSX 384 kb) Additional file 4: Table of transect locations (.xls). Locations of transects and fields surveyed in on-site portion of the study (May-June 2013). (XLSX 11 kb) Additional file 5: Map of all GPS tracked gulls’ locations (.pdf). (PDF 7882 kb) Additional file 6: Random effect of the tracked gull individuals for model 18 in Table 1 (.pdf). (PDF 134 kb) Additional file 7: Agricultural field parameters (.xls). Vegetation parameters of the agricultural fields surveyed in on-site portion of the study, along with *L. fuscus* presence/absence counts, earthworm, other gull species and wader numbers throughout the study period (May-June 2013). (XLSX 17 kb) Additional file 8: Proportion of land or sea or mixed foraging trips by GPS tracked gulls divided by year (.pdf). An extended version of Fig. 4 a. (PDF 850 kb)
