# Supplementary material for: Land or sea? Foraging area choice during breeding by an omnivorous gull
Source: Mov Ecol. 2016 May 15;4:11. doi: 10.1186/s40462-016-0078-5 (PMC4868019; doi:10.1186/s40462-016-0078-5)
Supplement: Additional file 1: — Table of lesser black-backed gulls GPS deployments (.doc). GPS deployments on lesser black-backed gulls at Stora Karlsö (2011–2013) with number of foraging trips per individual per year included in analysis. (PDF 125 kb) [file 40462_2016_78_MOESM1_ESM.pdf]

Additional file 1. Table of lesser black-backed gulls GPS deployments. GPS deployments on lesser black-backed gulls at Stora Karlsö (2011-2013) with number of foraging trips per individual per year included in analysis (see additional file 3 for raw data).

Ring Number	*Head-bill (mm)*	*Sex*	*Date tagged*	*Number of foraging trips per year*				
				2011	*2012*	*2013*	*Total*	
8114315	120.3	M	2011-05-21	46	105		151	
8114318	103	F	2011-05-25	46			46	‡
8114316	127	M	2011-05-22	27	2		29	
8114317	116.1	M	2011-05-23	14			14	‡
8114319	115.8	M	2011-05-25					*
8114314	104.5	F	2011-05-21	17	41	39	97	
8114320	121.4	M	2011-05-25	11			11	‡
8114321	118.5	M	2011-05-25	15			15	‡
8114312	-	F	2011-05-20	11			11	†‡
8114313	117.8	M	2012-05-21	56	105		161	
8111155	124.2	M	2012-06-01		52	59	111	
8111250	107.85	F	2012-06-03		47	88	135	
8114370	118.65	M	2012-05-31		36		36	
8111177	119.4	M	2012-05-31		37	56	93	
8114371	106.5	F	2012-06-05		63		63	
8111176	121	M	2012-06-05		51		51	
8114348	116.62	M	2013-06-01			61	61	
8114345	117.16	M	2013-05-27			69	69	
8114349	117.11	M	2013-06-03			22	22	
8114347	102.63	F	2013-06-01			33	33	
8114346	117.99	M	2013-05-31					*
			Total	243	539	427	1209	

* No data because of tag failure/ breeding abandonment

† Head-bill measurement not taken. Observed to be smaller member of pair, thus assumed female

‡ Not included in pre-laying analysis for 2012-2013 due to lack of data
