# Supplementary material for: Land or sea? Foraging area choice during breeding by an omnivorous gull
Source: Mov Ecol. 2016 May 15;4:11. doi: 10.1186/s40462-016-0078-5 (PMC4868019; doi:10.1186/s40462-016-0078-5)
Supplement: Additional file 2: — Detailed analytical methods on the processing of the GPS tracking data of the lesser black-backed gulls (.pdf). Information on which trips were included from the wider GPS tracking dataset and how activity within trips was classified between land and sea foraging is provided. (PDF 304 kb) [file 40462_2016_78_MOESM2_ESM.pdf]

Detailed methods: Processing of GPS tracking data

A. Criteria for inclusion of trips in analysis

The GPS data from the tracked lesser black-backed gulls (*Larus fuscus*) were initially segmented into trips based on a distance threshold around the nest of each tracked bird (see Methods in paper). As not all these trips were likely to be true foraging trips, or the quality of the data were poor for the trip (e.g. long gaps in GPS tracking), we first filtered the data using the following 6 criteria before then classifying trips according to their use of marine and terrestrial foraging (see section B below).

1. Exclusion of migratory trips

As the gulls were often followed across multiple years, the full GPS tracking data-set includes migratory activity. This was excluded by excluding trips that crossed a latitude of 40°N, all migratory trips crossed this latitude, but no trips during breeding did.

2. Number of GPS locations

Trips with <10 GPS locations were excluded. We consider that 10 GPS locations or more were sufficient for this analysis where detailed activity patterns are not analysed, the analysis focussing on a binary habitat use variable (land or sea foraging).

3. Trip duration

By examining a histogram of trip durations (figure 1) we decided to exclude trips of >2 days duration. Most trips were <2 days (95%), with longer multi-day trips mostly occurring either pre-breeding, or post-breeding/ pre-migration. Including overnight trips does risk inclusion of roosting activity, though this was avoided by classifying foraging habitat use based on foraging GPS locations only (see section B).

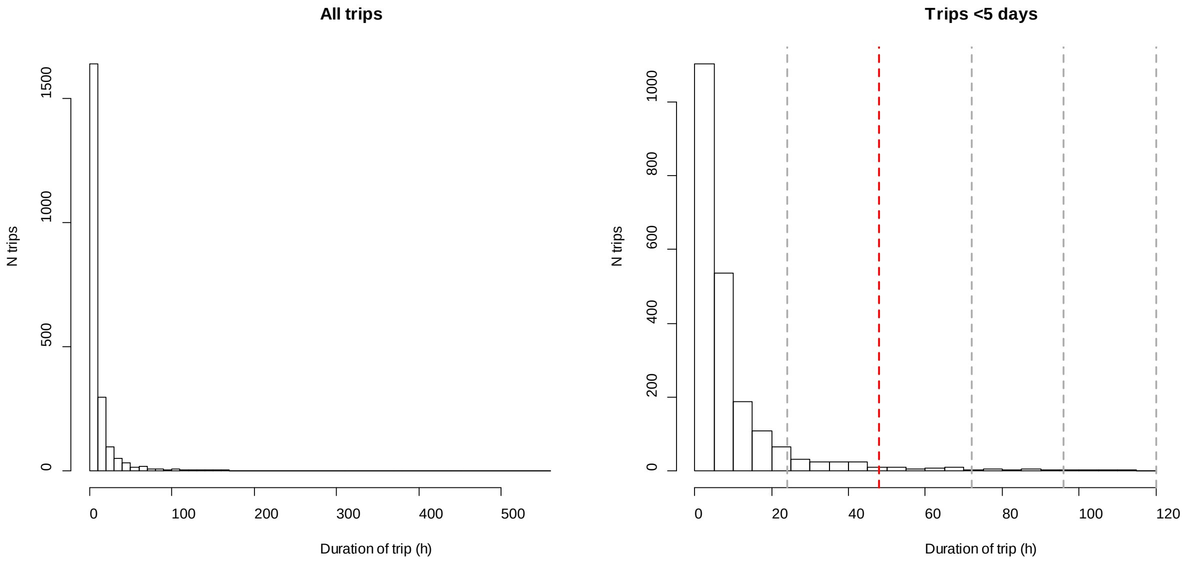

Figure 1. Duration of all trips after exclusion of migratory trips and those with <10 GPS fixes (left), trips of duration <5 days (right) with days indicated (grey broken lines), trips of <2 days (red broken line) were retained

4. Distance from colony

Following reference (Camphuysen et al. 2015) and inspecting our own data (figure 2) we considered trips where the maximum distance from colony <3 km to be non-foraging trips, a similar threshold (2 km) to

that used in (Corman et al. 2016); thus retaining trips fulfilling the above criteria (1-3) and where the maximum distance was >3 km.

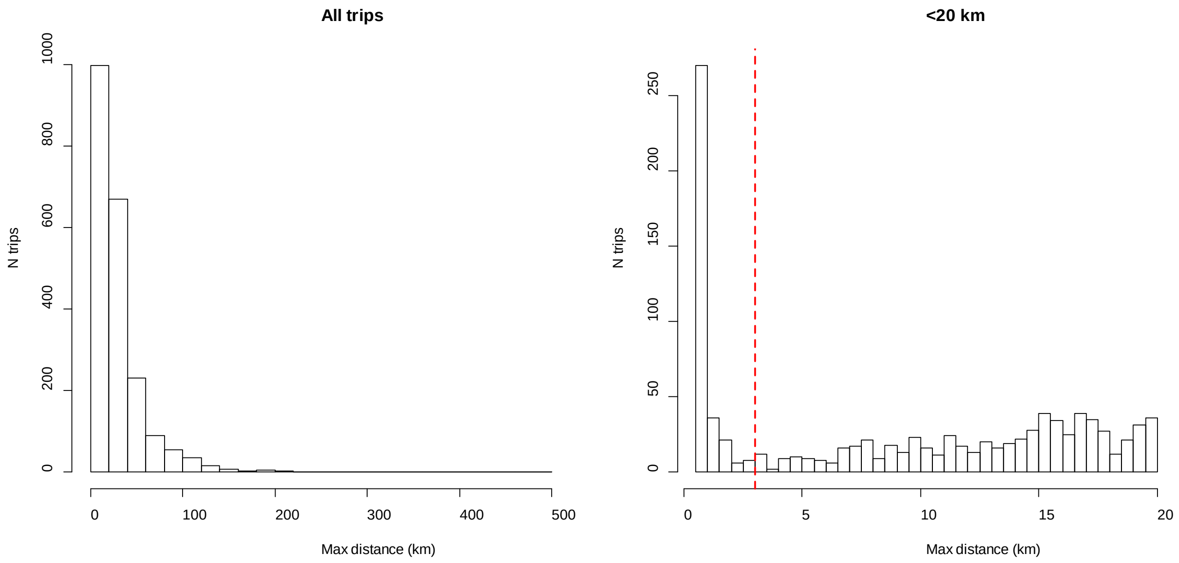

Figure 2. Distribution of maximum distance reached from the nest during all trips (left) after exclusion of trips fulfilling criteria 1-3 (above). Trips with a minimum distance of 3 km (red broken line, right) were retained for analysis

5. Time interval between GPS locations

Similarly to filtering criterion 2 we excluded trips where there were extended time gaps between GPS locations, to ensure we only included foraging trips with good information. Extended time gaps could arise for a number of reasons; poor GPS reception, battery depletion (more likely under extended overcast conditions where solar charging was greatly reduced), and when devices were configured to record GPS locations and/ or accelerometry data (not presented in this study) at high frequencies thus depleting the battery more rapidly. Following inspection of the distribution of the maximum duration of time gaps between GPS locations (figure 3) we excluded trips including gaps of 30 minutes or more.

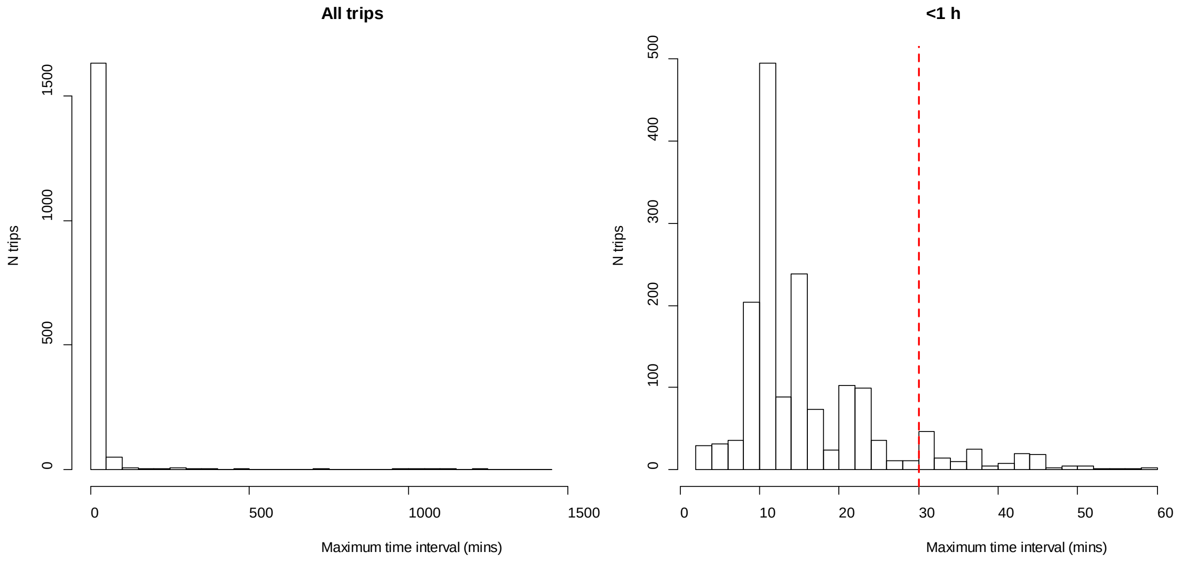

Figure 3. Distribution of maximum time interval between GPS location within a trip (left) after exclusion of trips fulfilling criteria 1-4 (above). Trips with a maximum time interval of 30 minutes (red broken line, right) or less were retained for analysis

6. Date period

Our study focussed on the core breeding period from incubation through to late chick-rearing, thus we only retained trips during this focal period (May 20th – July 21st) for the statistical analysis, though for reference pre-laying (April 30th – May 19th) is included in a supplementary figure (Additional file 7).

B. Foraging habitat use classification: land or sea?

Our analysis looks at the propensity of lesser black-backed gulls to use marine or terrestrial habitats during their foraging trips. After filtering trips to retain only foraging trips, and those with adequate data quality (see section A above) we then classified the proportion of these trips spent foraging on land or at sea. We achieved this by first resampling foraging trips to a common time interval, then labelling these derived GPS locations as foraging or non-foraging locations, then finally whether these were at sea or on land.

1. Interpolating GPS tracked trips to a common time interval

In order to standardize the classification of foraging trips as outlined in Calenge *et al.* (2009), we first interpolated the tracks to a common time interval of 300 s. Time intervals between GPS locations varied both within and between trips (figure 4). Within a trip, time interval could vary due to: variation in acquisition time (the time taken for the GPS unit to attain information from GPS satellites and calculate a position); missed locations, for example owing to shading leading to the unit not gaining a GPS location; and conditional device programs whereby log intervals may vary according to location or behaviour (ground speed). Between trips, log intervals varied mainly owing to differences in the device program.

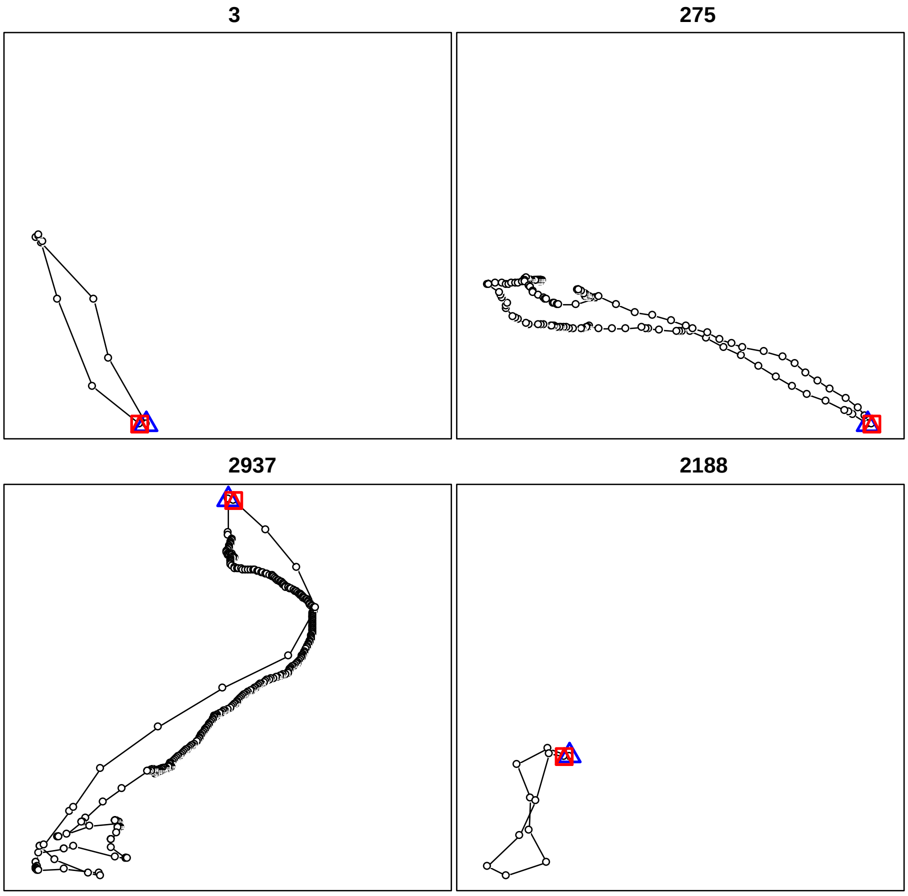

Figure 4. Four example GPS tracked foraging trips of lesser black-backed gulls. GPS time intervals were not necessarily regular during a foraging trip, nor consistent between trips. Thus data were interpolated to a common time interval (figure 5). Numbers refer to 'trip_id' (see additional file 3), gps locations (black open circles), and first (blue triangle) and final (red square) locations indicated

Trips were interpolated to a common time interval of 300s using function *redisltraj* in the R package *adehabitatLT* (Calenge *2006*), which led to trajectories with equal time intervals allowing habitat use and behaviour to be comparable between trips (cf. figures 5 and 4).

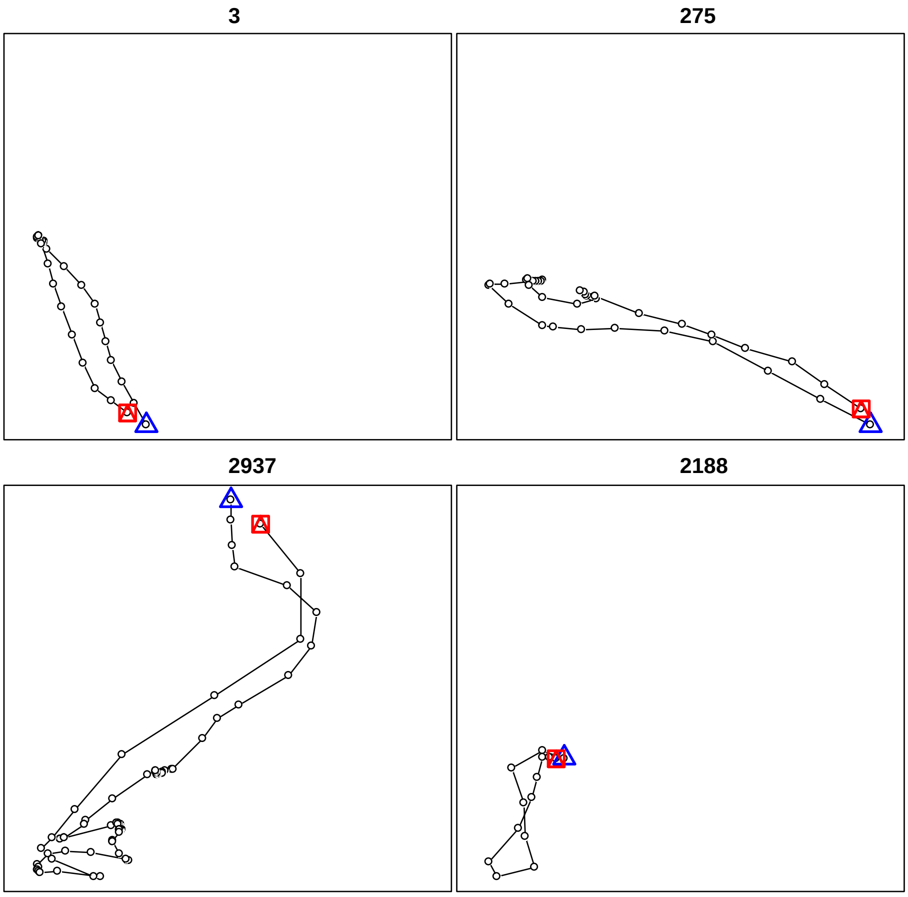

Figure 5. Foraging trips shown in figure 4 interpolated to a common time interval (300 s). Symbols as in figure 4

2. Classifying locations into foraging or non-foraging

Foraging locations were labelled through a combination of two speed thresholds (to exclude stationary and commuting/ directed flights) and including only locations over a minimum distance from the colony.

i. Speed thresholds

Foraging locations were considered to be those locations where the gulls were recorded moving at intermediate speeds, i.e. not stationary nor performing fast directed movements.

Locations where the step-length was >1.5 km (equating to a mean speed of >5 ms^{-1}) were considered to be mostly commuting/ directed flight (figure 6). During active area-restricted search at sea over 5 minutes (the time interval between locations) gulls would not be expected to achieve such high speeds which are only possible with relatively straight and sustained flights.

Likewise locations where the step-length was <200 m (<0.1 ms^{-1}) were considered to likely correspond to stationary activity (e.g. roosting, resting, or loafing), thus were not included as foraging locations (figure 6).

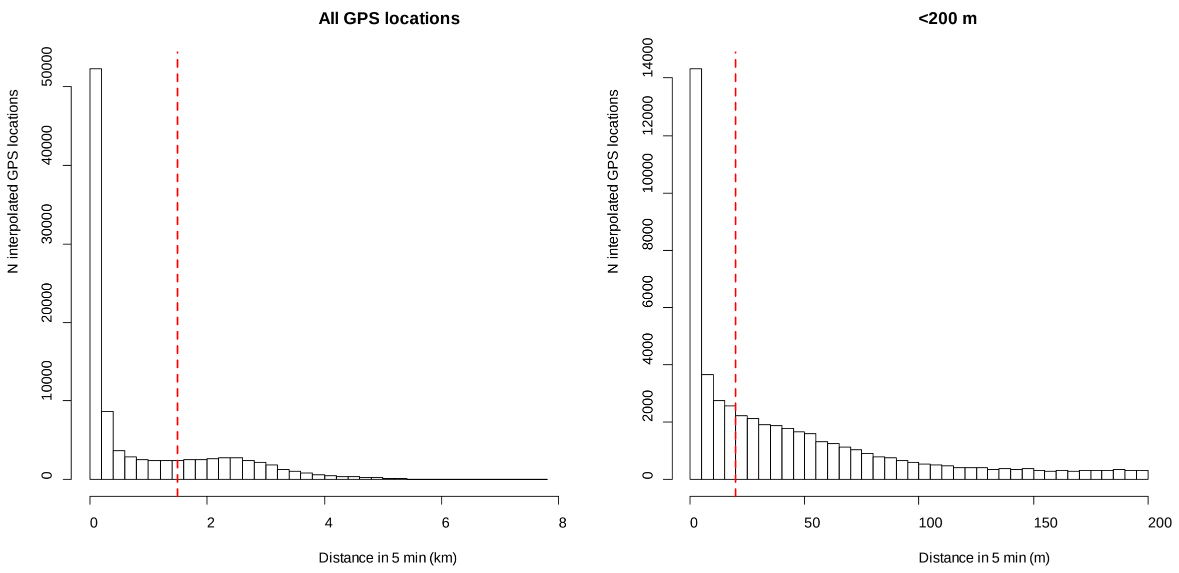

Figure 6. Straight-line distance covered between interpolated 300 s interval GPS locations, with a higher critical threshold (>1.5 km, red-dashed line, left) and lower critical threshold (<20 m, red-dashed line, right) set to exclude faster directed movements and stationary locations respectively from being classified as foraging locations

ii. Distance from colony

Locations very near the colony are likely to be non-foraging locations, thus locations <3 km from the colony were not included in foraging locations (figure 7).

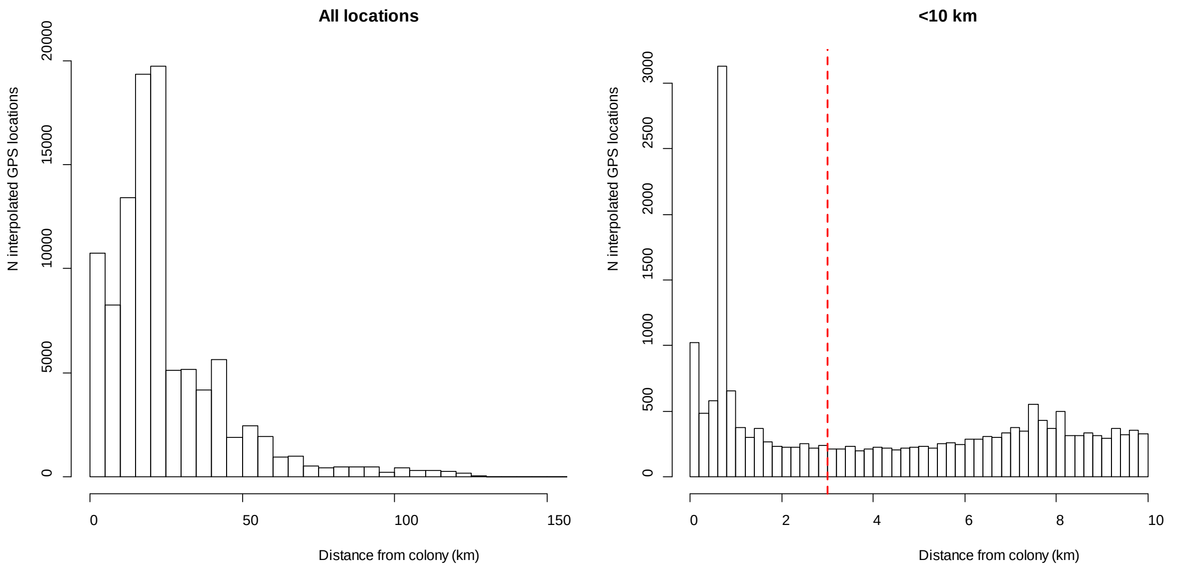

Figure 7. The distribution of distances from the breeding colony for all interpolated 300 s interval GPS locations (left), and those within 10 km of the colony (right). Locations close to the colony (<3 km, red-dashed line, right), were deemed to correspond to non-foraging activity

3. Calculating proportion of land foraging during trip

Foraging locations (classified according to the criteria above) were then labelled by habitat type, either sea or land. This was a simple criterion of whether the location was on land or at sea, using a polygon drawn around the only land foraging area (the island of Gotland) with a small buffer (see figure 8). The proportion of the foraging trip then corresponding to land based foraging, sea based foraging, or other activity (e.g. fast directed flight, stationary locations, or near the colony) was then obtained (see figure 2

in the main paper). For a small number of trips (14) no points were classified as foraging, as these were a small number of trips they were excluded from the analysis. For analysis the proportion of foraging time spent on land (variable *p_foraging_land* in additional file 3) was used. For illustration (figure 4 in the paper, and additional file 7 we also classified trips into three types (variable *trip_type* in additional file 3); land (>95 % of foraging locations on land), mix (5 – 95 % of foraging locations on land), and sea (<5 % of foraging locations on land).

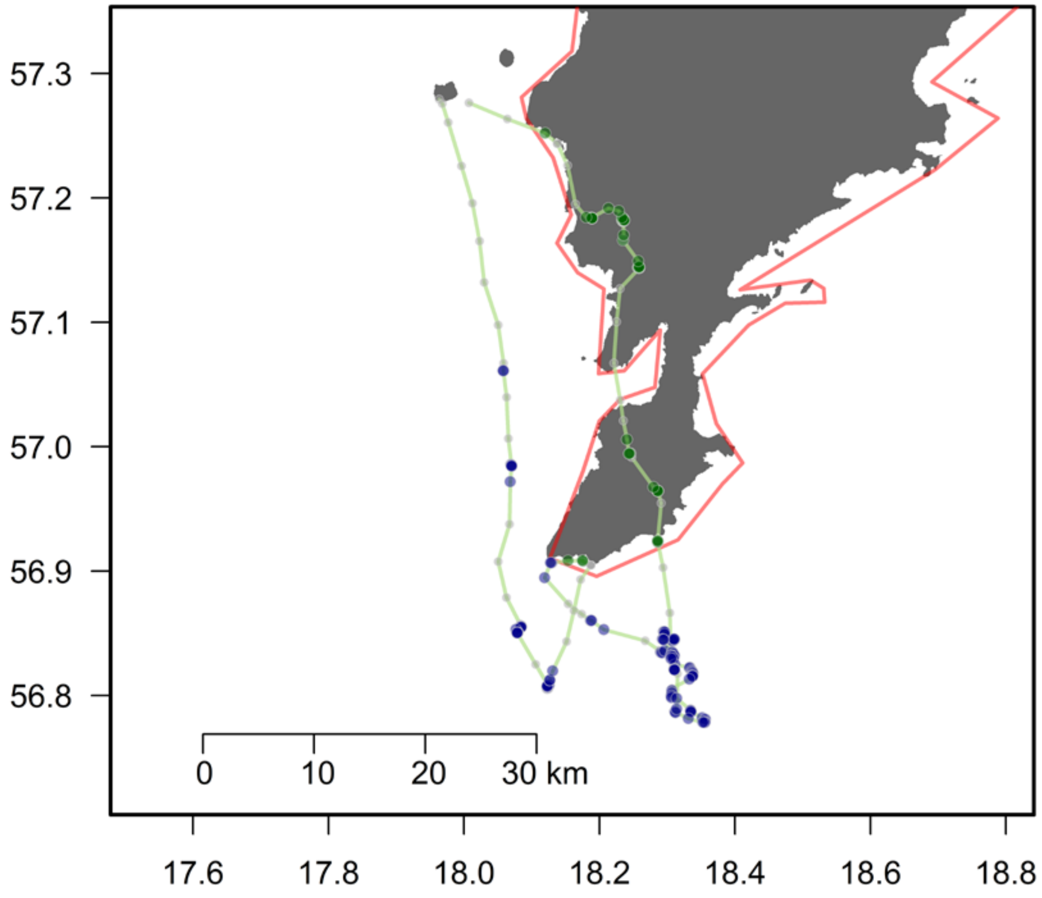

Figure 8. Foraging locations were classified as land (green filled circles) or sea (blue filled circles) according to whether they were within (land) or outside (sea) a polygon (red lines) following the coastline of the island of Gotland. Non foraging locations (grey filled small circles) were not classified

References

Calenge C. The package adehabitat for the R software: a tool for the analysis of space and habitat use by animals. Ecol Model. 2006; 197: 516-519.

Calenge C, Dray S, Royer-Carenzi M. The concept of animals' trajectories from a data analysis perspective. Ecol Inform. 2009; 4: 34–41.

Camphuysen KCJ, Shamoun-Baranes J, van Loon EE, Bouten W. Sexually distinct foraging strategies in an omnivorous seabird. Mar Biol. 2015; 162: 1417-1428.

Corman AM, Mendel B, Voigt CC, Garthe S. Varying foraging patterns in response to competition? A multicolony approach in a generalist seabird. Ecol Evol. 2016; doi: 10.1002/ece3.1884
