# Supplementary material for: Land or sea? Foraging area choice during breeding by an omnivorous gull
Source: Mov Ecol. 2016 May 15;4:11. doi: 10.1186/s40462-016-0078-5 (PMC4868019; doi:10.1186/s40462-016-0078-5)
Supplement: Additional file 5: — Map of all GPS tracked gulls’ locations (.pdf). (PDF 7882 kb) [file 40462_2016_78_MOESM5_ESM.pdf]

Additional file 5

All GPS positions (interpolated to regular 300 s intervals) for analyzed GPS trips (including pre-laying) excluding those positions <3 km from the colony location. Includes pre-laying and incubation (left) and chick-rearing (right). Coast-line is indicated (blue) over GPS locations. The density of locations (black circles) is illustrated by darker colors where more location symbols intersect. Utilization distributions were plotted with contours shown (red lines) for 25, 50, 75, 90, 95 % utilization distributions (with decreasing line-widths). Utilization distributions used a Brownian bridge movement model (Kranstauber *et al.* 2012), implemented with the *R* package *BBMM* (Nielson *et al.* 2013).

References:

Kranstauber, B., Kays, R., LaPoint, S.D., Wikelski, M., and Safi, K. (2012). A dynamic Brownian bridge movement model to estimate utilization distributions for heterogeneous animal movement. Journal of Animal Ecology.

Nielson, R. M., H. Sawyer, and T. L. McDonald (2013). BBMM: Brownian bridge movement model. BBMM: Brownian bridge movement model. http://CRAN.R-project.org/package=BBMM

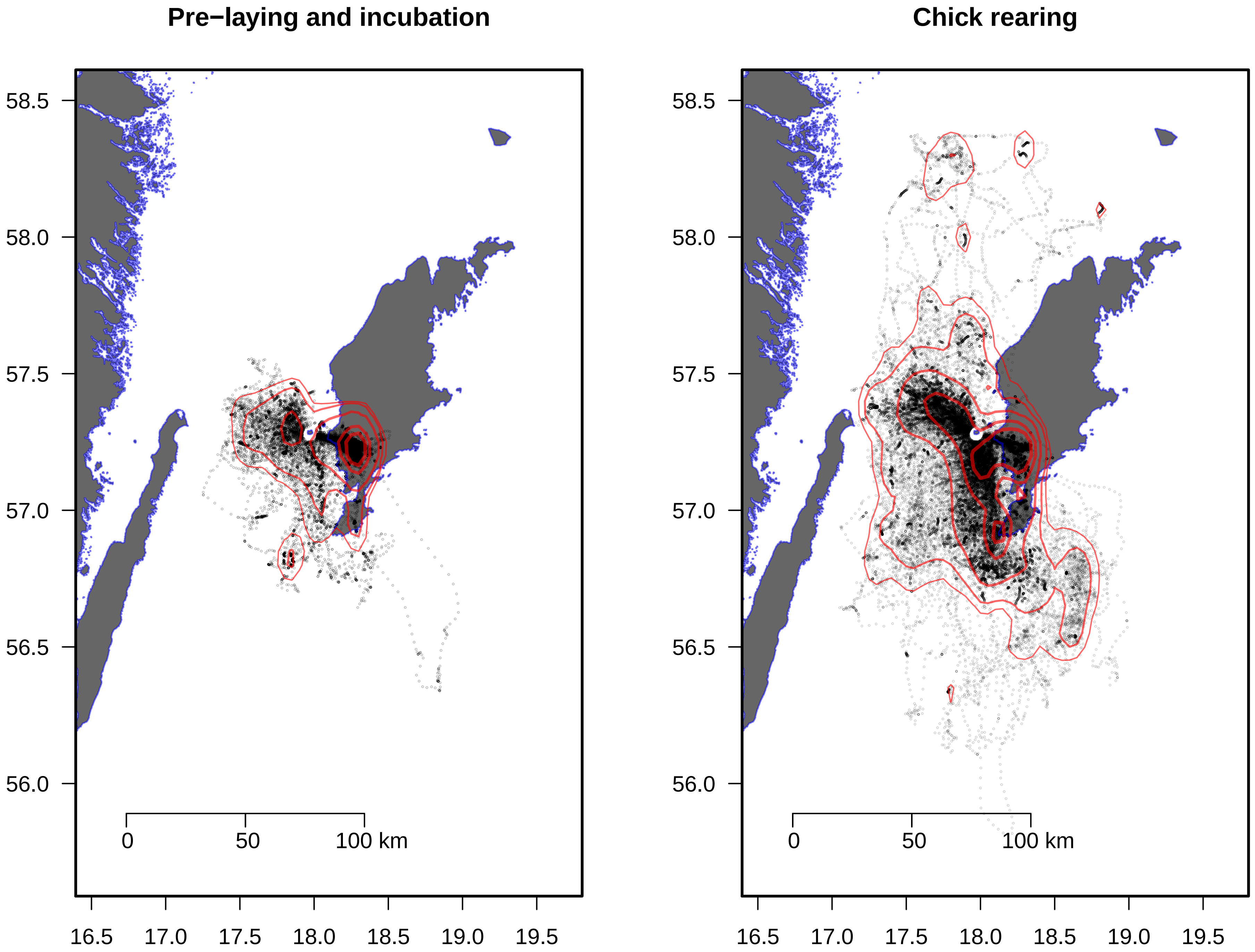

Pre−laying and incubation
Chick rearing
58.5
58.0
57.5
57.0
56.5
56.0
0
50
100 km
16.5
17.0
17.5
18.0
18.5
19.0
19.5
