# Supplementary material for: Land or sea? Foraging area choice during breeding by an omnivorous gull
Source: Mov Ecol. 2016 May 15;4:11. doi: 10.1186/s40462-016-0078-5 (PMC4868019; doi:10.1186/s40462-016-0078-5)
Supplement: Additional file 6: — Random effect of the tracked gull individuals for model 18 in Table 1 (.pdf). (PDF 134 kb) [file 40462_2016_78_MOESM6_ESM.pdf]

Additional file 6

Random effect terms of the individual tracked gull individuals for model 18 in Table 1. These show the increased (positive values) or decreased (negative values) probability of the 19 GPS tracked lesser black-backed gulls to forage on land for the finally selected statistical model together with 95% confidence intervals. Female (black) and male (red) gulls are indicated with colors.

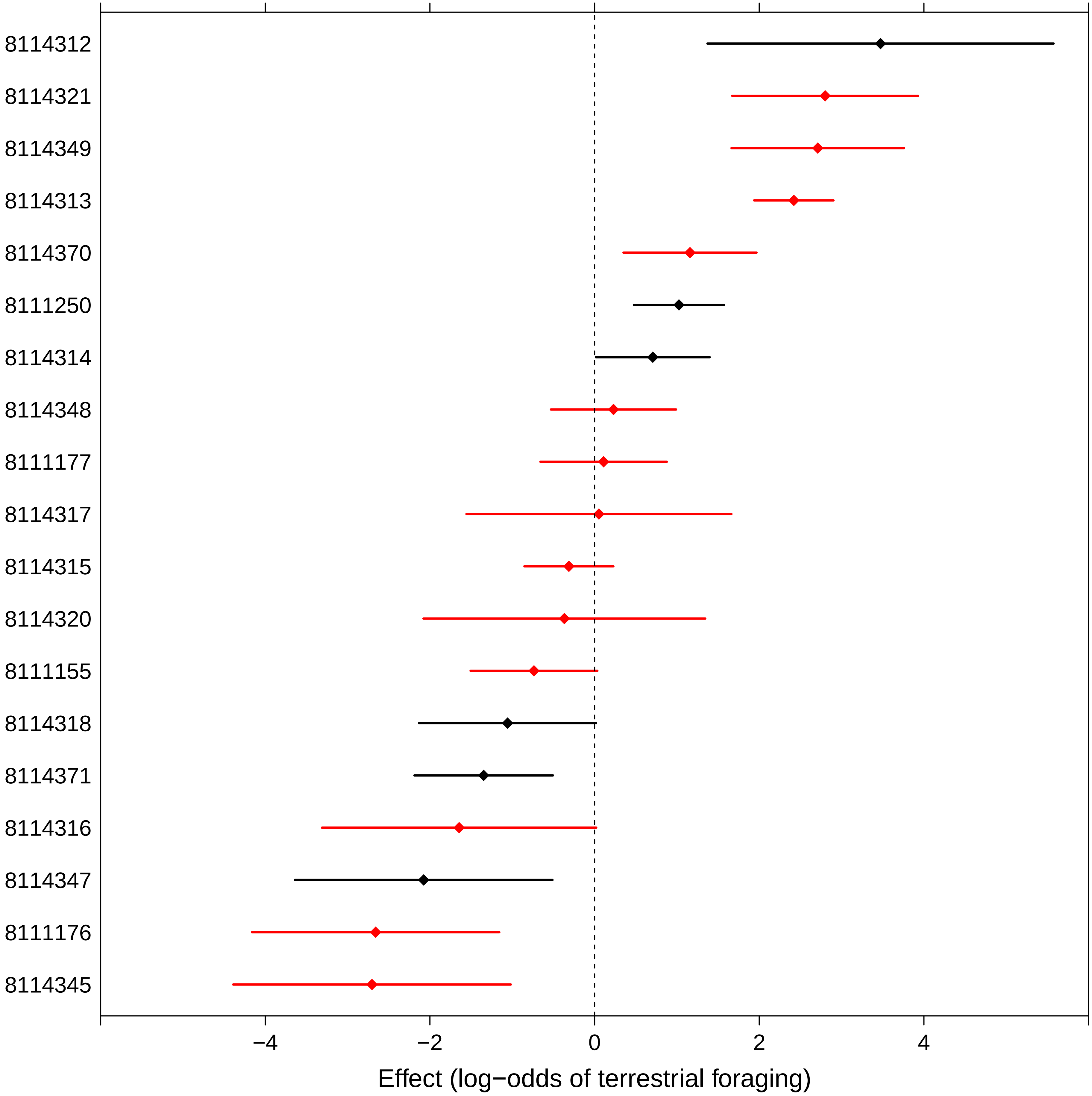
8114312
8114321
8114349
8114313
8114370
8111250
8114314
8114348
8111177
8114317
8114315
8114320
8111155
8114318
8114371
8114316
8114347
8111176
8114345
−4
−2
0
2
4
Effect (log−odds of terrestrial foraging)
