# Supplementary material for: Land or sea? Foraging area choice during breeding by an omnivorous gull
Source: Mov Ecol. 2016 May 15;4:11. doi: 10.1186/s40462-016-0078-5 (PMC4868019; doi:10.1186/s40462-016-0078-5)
Supplement: Additional file 8: — Proportion of land or sea or mixed foraging trips by GPS tracked gulls divided by year (.pdf). An extended version of Fig. 4 a. (PDF 850 kb) [file 40462_2016_78_MOESM8_ESM.pdf]

Additional file 8

Proportion of land (green), sea (blue), or mixed (magenta) foraging trips by GPS tracked gulls divided by year. Extended version of Fig 4 a (main paper), showing proportion of foraging trips of different classes through breeding period for 5-day periods. The breeding stages are indicated (vertical broken grey lines). Note in 2011 there were no pre-laying trips recorded, as the first gulls were tagged during incubation.

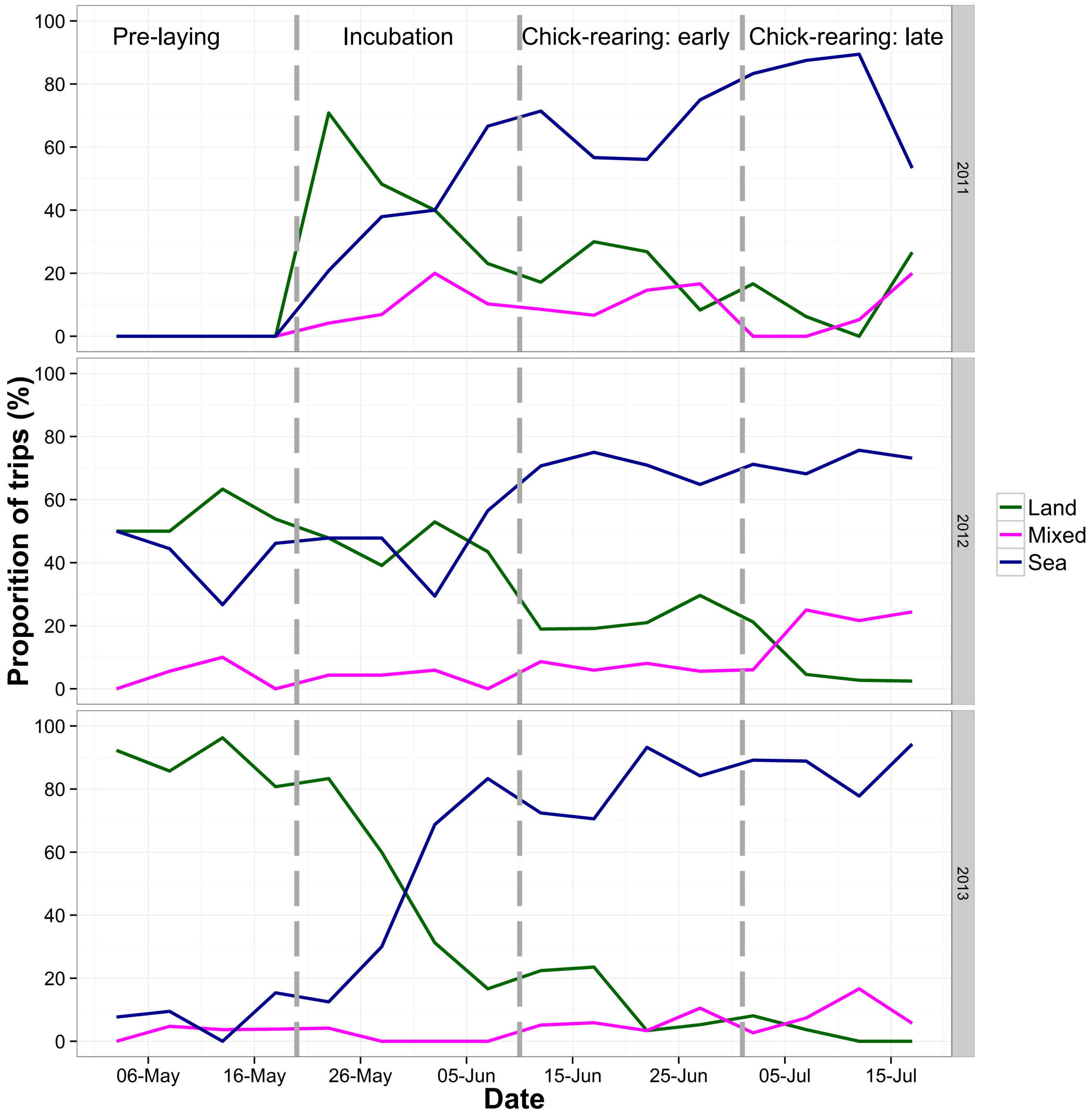
Pre-laying
Incubation
Chick-rearing: early
Chick-rearing: late
2011
2012
2013
Proportion of trips (%)
100
80
60
40
20
0
06-May
16-May
26-May
05-Jun
15-Jun
25-Jun
05-Jul
15-Jul
Date
Land
Mixed
Sea
